# Combined social determinants of health contributed to adverse health outcomes among depression: evidence from two national cohorts

**DOI:** 10.1017/S2045796025100176

**Published:** 2025-08-22

**Authors:** Xin Qi, Li Liu, Jin Yang, Chuyu Pan, Jingcan Hao, Wenming Wei, Shiqiang Cheng, Yifan Gou, Boyue Zhao, Yan Wen, Bolun Cheng, Feng Zhang

**Affiliations:** 1 Department of Psychiatry, The First Affiliated Hospital of Xi’an Jiaotong University, Xi’an, P. R. China; 2 Key Laboratory of Trace Elements and Endemic Diseases of National Health and Family Planning Commission, School of Public Health, Health Science Center, Xi’an Jiaotong University, Xi’an, P. R. China; 3 Precision Medicine Center, The First Affiliated Hospital of Xi’an Jiaotong University, Xi’an, P. R. China; 4 Department of Medical Oncology, The First Affiliated Hospital of Xi’an Jiaotong University, Xi’an, P. R. China; 5 Department of Medical Administration, The First Affiliated Hospital of Xi’an Jiaotong University, Xi’an, P. R. China

**Keywords:** cancer, cardiovascular disease, depression, health disparities, social determinants of health

## Abstract

**Aims:**

Social determinants of health (SDHs) exert a significant influence on various health outcomes and disparities. This study aimed to explore the associations between combined SDHs and mortality, as well as adverse health outcomes among adults with depression.

**Methods:**

The research included 48,897 participants with depression from the UK Biobank and 7,771 from the US National Health and Nutrition Examination Survey (NHANES). By calculating combined SDH scores based on 14 SDHs in the UK Biobank and 9 in the US NHANES, participants were categorized into favourable, medium and unfavourable SDH groups through tertiles. Cox regression models were used to evaluate the impact of combined SDHs on mortality (all-cause, cardiovascular disease [CVD] and cancer) in both cohorts, as well as incidences of CVD, cancer and dementia in the UK Biobank.

**Results:**

In the fully adjusted models, compared to the favourable SDH group, the hazard ratios for all-cause mortality were 1.81 (95% CI: 1.60–2.04) in the unfavourable SDH group in the UK Biobank cohort; 1.61 (95% CI: 1.31–1.98) in the medium SDH group and 2.19 (95% CI: 1.78–2.68) in the unfavourable SDH group in the US NHANES cohort. Moreover, higher levels of unfavourable SDHs were associated with increased mortality risk from CVD and cancer. Regarding disease incidence, they were significantly linked to higher incidences of CVD and dementia but not cancer in the UK Biobank.

**Conclusions:**

Combined unfavourable SDHs were associated with elevated risks of mortality and adverse health outcomes among adults with depression, which suggested that assessing the combined impact of SDHs could serve as a key strategy in preventing and managing depression, ultimately helping to reduce the burden of disease.

## Introduction

The social determinants of health (SDHs) encompass the environmental conditions in which individuals are born, reside, receive education, work, engage in leisure activities, worship and age (Spruce, 2019). These factors significantly influence a broad spectrum of health outcomes and contribute to disparities (Thornton *et al.*, 2016). Specifically, individuals with lower socioeconomic status experience roughly twice the incidence and mortality rates from cardiovascular disease (CVD) (Rosengren *et al.*, 2019). Evidence from the 2020 Lancet Commission on Dementia Prevention, Intervention, and Care indicated that 12 disadvantaged SDHs could account for approximately 40% of global dementia cases, and these SDHs may be preventable or delayable (Livingston *et al.*, 2020). Moreover, higher polysocial risk scores are linked to an elevated risk of health disparities, including CVD, dementia, type 2 diabetes and cancer (Javed *et al.*, 2021; Jou *et al.*, 2021; Kivipelto *et al.*, 2006; Zhao *et al.*, 2022). The Healthy People 2030 objectives propose a framework consisting of five domains – economic stability, education access and quality, healthcare access and quality, neighbourhood and built environment, and social and community context – highlighting the significance of SDHs in addressing health disparities.

Depression is the most common of psychiatric disorders worldwide, and approximately 280 million people suffer from depression (Yang *et al.*, 2021). Depression is the leading cause of years lived with disability since 2010, and psychiatric disorders account for the largest proportion of the global disease burden obtained by the Global Burden of Disease (GBD) study (Collaborators GMD, 2022; Liu *et al.*, 2020). Compared with the general population, in addition to a higher prevalence of suicide in depression (Bhak *et al.*, 2019), depression also contributes to higher risks of health disparities, including CVD (Meng *et al.*, 2020), cancer (Wang *et al.*, 2020) and dementia (Dafsari and Jessen, 2020; Yan *et al.*, 2024). Two prospective cohort studies involving Chinese adults found that depression significantly raises the risk of CVD mortality, particularly among men in the multivariable-adjusted models (Meng *et al.*, 2020). Moreover, depression is notably linked to an increased risk of cancer incidence, cancer-specific mortality and poorer survival outcomes, although reverse causality may also be a factor (Wang *et al.*, 2020). Additionally, comparing individuals without depression or cognitive impairment, those with depression exhibited a higher risk of developing subsequent dementia, with a hazard ratio (HR) of 1.65 (Yan *et al.*, 2024).

The widening health disparities among patients with depression have been driven by social and environmental conditions that act as SDHs, and the relationship between SDHs and depression has been extensively documented (Kammer-Kerwick *et al.*, 2024). For example, lower educational attainment is associated with an increased risk for depression in various countries, which may be attributed to the stress linked to lower socioeconomic status, less effective coping strategies or unhealthier lifestyles (Chlapecka *et al.*, 2020; Peyrot *et al.*, 2015). Individuals with the lowest incomes are typically 1.5 to 3 times more likely than those with the highest incomes to experience depression (Liu *et al.*, 2023; Ridley *et al.*, 2020). Furthermore, low income could lead to greater exposure to trauma, violence and crime, as well as lower social status, further impacting depression (Ridley *et al.*, 2020). On the other hand, social participation plays an effective role in mediating emotional social support for depression among older adults (Choi *et al.*, 2021). Although extensive research has explored the impact of socioeconomic status-related factors on depression, prior investigations have mainly focused on the effects of single SDH on the adverse outcomes in depression and rarely leveraged large-scale national cohorts to analyse how combined SDHs influence cause-specific mortality (e.g., CVD and cancer) and disease progression (e.g., dementia) among individuals with depression in this vulnerable population (Liu *et al.*, 2025; Rajan *et al.*, 2020). Crucially, the impact of combined SDHs on adverse health outcomes and mortality in depression remains underexplored, with limited empirical evidence quantifying their aggregate risk.

To address these gaps, this study utilizes data from two national cohort studies (UK and USA) to systematically investigate the associations between combined SDHs and (1) mortality risk (all-cause, CVD and cancer) among participants with depression in the UK and USA, and (2) the incidence of health outcomes (CVD, cancer and dementia) among participants with depression in the UK cohort. By focusing on the impact of combined SDHs on health outcomes among individuals with depression, this research aims to inform targeted interventions to reduce health disparities in depression.

## Materials and methods

### Study design and participants

**Figure 1.**
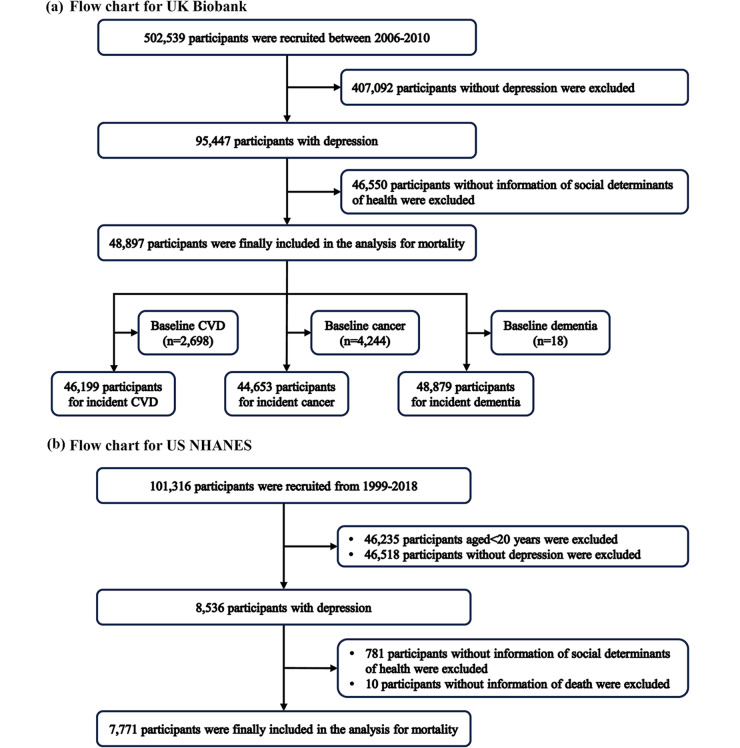
Flowchart for the selection of the study population in the UK Biobank (a) and the US NHANES (b) cohorts. Abbreviations: NHANES, National Health and Nutrition Examination Survey; CVD, cardiovascular disease.

Two national cohorts were used in this study. In the UK Biobank study, more than 500,000 participants aged 40–69 years were recruited between 2006 and 2010 and were from 22 follow-up assessments (Sudlow *et al.*, 2015). Participants with depression were identified based on self-reported (field ID: 20002, 1,286), Patient Health Questionnaire (PHQ)-9 score ≥ 5 and International Classification of Diseases-10 (ICD-10, field ID: 41270 and 41280, F32 to F33). PHQ-9 is a classification algorithm for measuring depression severity with a total score of 0–27 and is based on nine depressive symptoms and signs (field IDs: 20507, 20508, 20510, 20511, 20513, 20514 and 20517–20519) (Kroenke *et al.*, 2010). Participants with depression at baseline were included in the analysis of life expectancy and mortality (Fig. 1a). Participants without information on SDHs were excluded. Besides, for the analysis of incident outcomes, we excluded the participants with outcomes of interest at baseline (Fig. 1a).

The US National Health and Nutrition Examination Survey (NHANES) study was conducted by the Centers for Disease Control and Prevention (CDC) and the National Health Statistics Center, and was an ongoing annual survey from 1999. Detailed information on population and methodology is available at the NHANES website (www.cdc.gov/nchs/nhanes, accessed on 4 September 2024). Individuals with depression were identified by the Composite International Diagnostic Interview from 1999 to 2004 and PHQ-9 ≥ 5 from 2005 to 2019. We excluded the individuals without information on SDHs and follow-up (Fig. 1b).

### Assessment of SDHs

The SDH variables selected in this study were based on Healthy People 2030 objectives and the previous study (Zhong *et al.*, 2024). Different SDH variables were selected in UK Biobank and US NHANES studies due to different study designs, and detailed information on five SDH domains in this analysis is shown in Supplementary Table S1. Regarding financial circumstances, both cohorts took into account household income and employment status; the UK Biobank further included area-level income deprivation, while the US NHANES additionally considered food security. Regarding education access and quality, both cohorts accounted for educational attainment, with the UK Biobank also incorporating area-level education deprivation. Regarding healthcare access and quality, UK Biobank assessed area-level healthcare deprivation, whereas the US NHANES focused on healthcare access and health insurance coverage. Regarding neighbourhood and built environment, both cohorts considered accommodation stability, while the UK Biobank additionally considered area-level crime scores and the natural environment. Regarding social and community context, the UK Biobank incorporated living alone or with partners, social support, social activity, social isolation and emotional distress, whereas the US NHANES considered race and marital status. Area-level data were derived from the index of multiple deprivation scores based on the Lower-layer Super Output Area in the UK Biobank, with all other information collected through questionnaires (Supplementary Table S1).

A weighted combined SDH score was constructed to account for varied magnitudes of the associations between different SDHs and health outcomes, and this approach has been used in epidemiological analyses (Lourida *et al.*, 2019; Zhong *et al.*, 2024). Each SDH was divided into advantaged and disadvantaged levels (Supplementary Table S1). Cox regression model for all-cause mortality was used to calculate β coefficients of each SDH (comparing disadvantaged to advantaged level) after adjustment of age, sex, body mass index (BMI), smoking status, drinking status, physical activity, diet, and prevalence of hypertension and diabetes (Supplementary Tables S2 and S3). The combined scores of SDHs were calculated as the sum of the weighted scores for each SDH according to β coefficients of each SDH in both cohorts (Supplementary Tables S2 and S3). An unweighted SDH score was also constructed by 0 point representing the advantaged level and 1 point for the disadvantaged levels of each SDH in a sensitivity analysis. The total combined and unweighted SDH scores ranged from 0 to 14 in the UK Biobank and from 0 to 9 in the US NHANES. Higher combined SDH scores indicate less favourable SDHs. Participants were then categorized into three groups by tertiles, with the bottom, middle and top thirds corresponding to the favourable, medium and unfavourable SDH groups, respectively.

### Measurements of covariates

A range of important covariates were collected in this analysis, including age, sex (male and female), BMI, lifestyle behaviours (smoking status, drinking status, physical activity and diet) (Zhong *et al.*, 2024), and history of hypertension and diabetes. Specifically, smoking status and drinking status were classified as never, previous or current. Physical activity was categorized as inactive group, insufficiently active group and active group according to the spent time of performing walking, moderate and vigorous activity. Diet was classified as a healthy diet (above the median) and an unhealthy diet (below the median) based on the Healthy Eating Index in the US NHANES and a dietary recommendation according to a previous study in the UK Biobank (Li *et al.*, 2020; Zheng *et al.*, 2023). In the UK Biobank, the prevalence of hypertension was defined based on ICD-10 codes from I10 to I50, and diabetes was diagnosed through ICD-10 codes E10–E14. In the US NHANES, hypertension was defined based on one or more of these conditions: the use of antihypertensive medication, systolic blood pressure ≥ 140 mmHg, diastolic blood pressure ≥ 90 mmHg or the response to the question ‘Ever told you had high blood pressure’. Diabetes was defined based on one or more of these conditions: glycosylated haemoglobin (HbA1c) ≥ 6.5%, the current use of diabetes medication or insulin, or the response to the question ‘doctor told you have diabetes’. The detailed information of covariates was documented in Supplement 1 and Supplementary Table S4.

For missing covariates, linear regression models were used to impute continuous variables and logistic regression models were used to impute categorical variables through R ‘Mice’ packages. Multiple imputations by chained equations with five imputations were used to impute the missing values of covariates. The percentages of missing values for covariates in the UK Biobank and the US NHANES cohorts are shown in Supplementary Table S5.

### Definitions of outcomes

Mortality (including all-cause, CVD and cancer) and incident non-fatal outcomes (CVD, dementia and cancer) were identified using ICD-10 codes (Supplementary Tables S6 and S7). Through the National Death Index and the National Health Service Information Center, deaths were ascertained to 31 December 2019 in the US NHANES and UK Biobank cohorts.

The specific sources of incident non-fatal outcomes were provided in Supplementary Table S7, which were only available in the UK Biobank. To identify participants without non-fatal outcomes, both self-reported and hospital inpatient data were utilized. Hospital inpatient data mapped to ICD-10 codes, death records and follow-up loss data were employed to identify participants with incident non-fatal outcomes. For participants free of outcomes of interest at baseline, their follow-up time ended on the date of the first diagnosis of outcomes, the date of death (field ID: 40000), the date of loss to follow-up (field ID: 191) or the date of the end of current follow-up (31 December 2019), whichever occurred first.

### Statistical analysis

Separate analyses for the two cohorts were performed in this study. Baseline characteristics were described across combined SDH groups. For continuous variables, means ± standard errors or medians with interquartile ranges were calculated and differences across three groups were tested through ANOVA tests when data were normally distributed and homogeneity of variance; otherwise, Kruskal–Wallis *K* tests were used. For categorical variables, frequencies (percentages) were calculated, and differences across groups were tested by the 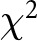 test.

Cox proportional hazards regression models were used to estimate HRs and 95% confidence interval (CI) of combined SDHs on the risk of mortality (including all-cause, CVD and cancer) and non-fatal outcomes (including the incidence of CVD, cancer and dementia) among adults with depression. Person-years were calculated from the date of recruitment to the date of the first diagnosis of outcomes, death, loss to follow-up or the end of follow-up (31 December 2019), whichever occurred first. Two models were performed in this study: Model 1 adjusted for sex and age; Model 2 additionally adjusted for BMI, smoking status, drinking status, physical activity, diet, and prevalence of hypertension and diabetes. Survival over time was estimated using the Kaplan–Meier curve, and the log-rank test was employed to assess differences in survival curves among the three combined SDH groups.

To assess the robustness across different subgroups, we conducted subgroup analyses for age (<60 years and ≥60 years), sex (female and male), BMI (<25 kg/m^2^, 25–29.9 kg/m^2^ and ≥30 kg/m^2^), smoking status (never, previous and current), drinking status (never, previous and current), physical activity (inactive, insufficiently active and active physical activity group), diet (unhealthy and healthy), hypertension (yes and no) and diabetes (yes and no). Interaction terms between the combined SDH score and subgroup variables were included in the model to examine differences between subgroups. Only individuals who were free of the corresponding disease at baseline were included in the analysis for incident diseases. The models used in the subgroup analyses were adjusted for the same covariates as Model 2, except for the stratification variable which was used for stratification purposes.

In addition, several sensitivity analyses were conducted to validate the robustness of findings. First, we excluded the participants with CVD and cancer at baseline in the two cohorts to reduce the possibility of reverse causation. Second, participants who experienced outcomes of interest within a follow-up period of less than 2 years were excluded. Third, an unweighted SDH score was also performed to assess the robustness of the results. Fourth, to account for competing risks, Fine–Gray subdistribution hazards models were additionally performed, treating cancer death as a competing event in CVD mortality analyses and CVD death in cancer mortality analyses.

All of data cleaning and analyses were conducted in R 4.1.2. Two-sided *P* values of <0.05 were considered statistically significant. A Bonferroni-corrected threshold of *P* < 2.27 × 10^−3^ (0.05/22) was applied to determine significance in subgroup analyses.

## Results

### Baseline characteristic

In the UK Biobank, 95,447 participants were identified with depression among those 46,550 participants without SDH data, and 48,897 participants (36.9% male) with a median age of 55 years were finally included in the analysis for mortality (Supplementary Table S8). In the US NHANES, we included 8,536 participants with depression among those 791 participants without SDH and death data, and there are 7,771 participants (39.6% male) with a median age of 48 years finally included in the analysis for mortality (Supplementary Table S8).

Participants in unfavourable SDH group were more likely to be female, have a higher BMI level, have a greater prevalence of hypertension and diabetes, and exhibit unhealthy lifestyle behaviours, including smoking and unhealthier dietary habits. Additionally, in the US NHANES, they tended to be older and less willing to engage in physical activity. Baseline characteristics of the study population grouped by combined SDHs in the UK Biobank and US NHANES cohorts were presented in Supplementary Table S8. The percentage of participants with disadvantaged levels for each SDH is shown in Supplementary Table S9.

### The influence of combined SDHs on the mortality of depression

During a median follow-up of 10.44 years, 1,683 deaths were recorded in the UK Biobank, among which 215 deaths were from CVD and 543 deaths were from cancer (Table 1). In the US NHANES, 943 deaths were documented during a median follow-up of 7.25 years, with 253 deaths from CVD and 208 deaths from cancer (Table 1).

**Table 1.** Associations between the combined SDHs and mortality among adults with depression in the UK Biobank and US NHANES cohorts

	UK Biobank cohort					US NHANES cohort				
	Favourable	Medium	Unfavourable		*P*-trend	Favourable	Medium	Unfavourable		*P*-trend
Weighted SDH Score Range	[0–2.84]	(2.84–4.91]	(4.91–14]			[0–4.2]	(4.2–6.6]	(6.6–9.0]		
All-Cause Mortality										
Number of participants	16,442	16,158	16,297			2,601	2,649	2,521		
Number of cases; person-years	408; 173,224	437; 169,580	838; 169,601			129; 21,253	347; 19,515	467; 17,811		
HR (95% CI) in Model 1	1 (Ref)	1.08 (0.95–1.24)	2.22 (1.97–2.5)		<2 × 10^−16^	1 (Ref)	1.82 (1.48–2.24)	2.78 (2.28–3.40)		<2 × 10^−16^
HR (95% CI) in Model 2	1 (Ref)	1.02 (0.89–1.17)	1.81 (1.60–2.04)		<2 × 10^−16^	1 (Ref)	1.61 (1.31–1.98)	2.19 (1.78–2.68)	1.01 × 10^−14^	
Cardiovascular disease mortality										
Number of cases	42	48	125			27	100	126		
HR (95% CI) in Model 1	1 (Ref)	1.18 (0.78–1.79)	3.33 (2.35–4.73)	1.90 × 10^−13^		1 (Ref)	2.18 (1.41–3.36)	3.15 (2.06–4.82)		3.05 × 10^−8^
HR (95% CI) in Model 2	1 (Ref)	1.09 (0.72–1.64)	2.57 (1.80–3.68)	1.12 × 10^−8^		1 (Ref)	1.91 (1.24–2.96)	2.42 (1.56–3.73)		7.66 × 10^−5^
Cancer mortality										
Number of cases	140	156	247			43	75	90		
HR (95% CI) in Model 1	1 (Ref)	1.09 (0.87–1.37)	1.84 (1.50–2.27)		1.71 × 10^−9^	1 (Ref)	1.17 (0.80–1.72)	1.64 (1.13–2.39)		5.30 × 10^−3^
HR (95% CI) in Model 2	1 (Ref)	1.04 (0.83–1.31)	1.55 (1.26–1.92)		2.01 × 10^−5^	1 (Ref)	1.06 (0.72–1.56)	1.39 (0.95–2.05)		6.27 × 10^−2^

*Note:* Model 1 was adjusted for age and sex; Model 2 was additionally adjusted for BMI, smoking status, drinking status, diet, physical activity, and prevalence of hypertension and diabetes.

Abbreviations: SDH, social determinant of health; NHANES, National Health and Nutrition Examination Survey; HR, hazard ratio; CI, confidence interval.

After multivariable adjustment (Model 2), compared to the favourable SDH group, the HRs for all-cause mortality were 1.02 (95% CI: 0.89–1.17) in the medium SDH group and 1.81 (95% CI: 1.60–2.04) in the unfavourable SDH group in the UK Biobank cohort. In the US NHANES cohort, the HR for the medium SDH group was 1.61 (95% CI: 1.31–1.98) and for the unfavourable SDH group was 2.19 (95% CI: 1.78–2.68). Survival curves by three combined SDH groups in the UK Biobank and the US NHANES are shown in Supplementary Figure S1.

Furthermore, higher unfavourable SDHs were significantly associated with increased mortality risks for both CVD and cancer in the UK Biobank cohort (*P*-trend < 0.05). In the US NHANES cohort, this association remained significant for CVD mortality but not for cancer mortality after full adjustment (Table 1). Specifically, for CVD mortality, the HRs of the unfavourable SDH group in Model 2 were 2.57 (95% CI: 1.80–3.68) in the UK Biobank cohort and 2.42 (95% CI: 1.56–3.73) in the US NHANES cohort. For cancer mortality, the HRs of the unfavourable SDH group in Model 2 were 1.55 (95% CI: 1.26–1.92) in the UK Biobank cohort and 1.39 (95% CI: 0.95–2.05) in the US NHANES cohort.

### The influence of combined SDHs on incident diseases of depression

In the UK Biobank cohort, 2,950 participants experienced incident CVD during a median follow-up of 10.39 years, while 4,653 participants developed incident cancer during a median follow-up of 10.38 years. Additionally, 331 participants were diagnosed with incident dementia during a median follow-up of 10.44 years.

In the fully adjusted model (Model 2), with the favourable SDH group as reference, the HR for incident CVD was 1.12 (95% CI: 1.02–1.23) in the medium SDH group and was 1.19 (95% CI: 1.08–1.30) in the unfavourable SDH group. For incident dementia, the HR was 1.51 (95% CI: 1.11–2.06) in the medium SDH group and 2.10 (95% CI: 1.56–2.83) in the unfavourable SDH group. Regarding incident cancer, the unfavourable SDH group showed a higher risk (HR = 1.12, 95% CI: 1.03–1.21) compared to the favourable SDH group in Model 1; however, this association was not statistically significant (HR = 1.01, 95% CI: 0.94–1.10) after multivariable adjustment (Table 2).

**Table 2.** Associations between the combined SDHs and incident diseases among adults with depression in the UK Biobank cohort

	UK Biobank cohort			
	Favourable	Medium	Unfavourable	*P*-trend
Weighted SDH score range	[0–2.84]	(2.84–4.91]	(4.91–14]	
Cardiovascular disease				
Number of participants	15,872	15,362	14,965	
Number of cases; person-years	858; 163,415	1,006; 157,035	1,087; 151,410	
HR (95% CI) in Model 1	1 (Ref)	1.23 (1.13–1.35)	1.46 (1.34–1.60)	<2 × 10^−16^
HR (95% CI) in Model 2	1 (Ref)	1.12 (1.02–1.23)	1.19 (1.08–1.30)	2.91 × 10^−4^
Cancer				
Number of participants	15,136	14,774	14,743	
Number of cases; person-years	1,209; 154,703	1,215; 150,461	1,286; 149,094	
HR (95% CI) in Model 1	1 (Ref)	1.01 (0.93–1.09)	1.12 (1.03–1.21)	5.76 × 10^−3^
HR (95% CI) in Model 2	1 (Ref)	0.97 (0.90–1.06)	1.01 (0.94–1.10)	7.21 × 10^−1^
Dementia				
Number of participants	16,438	16,154	16,287	
Number of cases; person-years	65; 173,026	111; 169,316	155; 169,086	
HR (95% CI) in Model 1	1 (Ref)	1.62 (1.19–2.20)	2.51 (1.88–3.36)	1.48 × 10^−10^
HR (95% CI) in Model 2	1 (Ref)	1.51 (1.11–2.06)	2.10 (1.56–2.83)	5.18 × 10^−7^

*Note:* Model 1 was adjusted for age and sex; Model 2 was additionally adjusted for BMI, smoking status, drinking status, diet, physical activity, and prevalence of hypertension and diabetes.

Abbreviations: SDH, social determinant of health; NHANES, National Health and Nutrition Examination Survey; HR, hazard ratio; CI, confidence interval.

### Subgroup analyses

**Figure 2.**
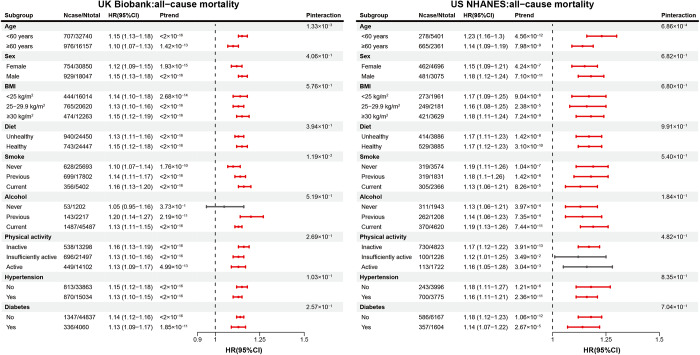
Subgroup analyses of the associations between combined SDH scores and all-cause mortality among adults with depression in the UK Biobank and US NHANES cohorts. The red points and lines indicate significant results, while the grey represents non-significant findings. Abbreviations: SDHs, social determinants of health; NHANES, National Health and Nutrition Examination Survey; HR, hazard ratio; CI, confidence interval; BMI, body mass index.

In different subgroups, higher unfavourable SDH scores were associated with increased risks of all-cause mortality among adults with depression in both the UK Biobank and US NHANES cohorts (most *P* interaction > 0.05, Fig. 2). Specifically, higher unfavourable SDH scores were associated with increased all-cause mortality risk among young adults, males, individuals with obesity, physcially inactive persons, and those without hypertension or diabetes. Additionally, except for incident cancer, most of the associations between SDHs and incident CVD and incident dementia remained consistent and statistically significant in subgroup analysis (Supplementary Fig. S2).

### Sensitivities analyses

The sensitivity analyses largely supported the findings of the main analysis (Supplementary Tables S10–S13). When excluding participants with CVD and cancer at baseline, the association between combined SDHs and all-cause mortality was even stronger in the unfavourable SDH group compared to the favourable SDH group (HR = 2.29, 95% CI: 1.74–3.02) in the US NHANES cohort (Supplementary Table S10). Similarly, when excluding participants within 2 years of follow-up time, the associations of combined SDHs with CVD mortality and incident CVD were stronger in the unfavourable SDH group compared to the favourable SDH group (HR = 2.74, 95% CI: 1.85–4.07 for CVD mortality; HR = 1.22, 95% CI: 1.10–1.35 for incident CVD) in the UK Biobank cohort (Supplementary Table S11). The findings for the unweighted SDH score were largely consistent with the main analysis using the combined SDH score (Supplementary Table S12). Fine–Gray subdistribution hazards models yielded directionally consistent associations with the primary Cox models, though with marginally attenuated effect sizes (Supplementary Table S13).

## Discussion

In this study, we constructed a combined SDH score to investigate the impact of SDH burden on mortality and adverse health outcomes among individuals with depression. Based on data from two national cohorts, our findings demonstrated consistent associations between combined SDHs and mortality, as well as additional health risks, in adults with depression across various social contexts. Compared to the favourable SDH group, unfavourable and medium SDH groups were associated with increased risks of death and the occurrence of diseases, including incident CVD, incident cancer and incident dementia among adults with depression, as well as consistent associations across different subgroups.

The impact of SDHs on mortality in individuals with depression is significantly greater than that observed in the general population. Studies have shown that multiple SDHs, such as income, social isolation and loneliness, increase the risk of all-cause mortality (Brandão *et al.*, 2019; Motillon-Toudic *et al.*, 2022; Zhou *et al.*, 2024). Notably, the individuals with depression living in low- and middle-income countries are found to be associated with excess mortality among the elderly (Brandão *et al.*, 2019). In addition to a single social factor, there is a growing interest in emerging literature to explore the impact of comprehensive social factors on depression (Pan *et al.*, 2023). Furthermore, research showed that as the level of disadvantaged SDHs increases, there is a corresponding rise in mortality within the general population (Holt-Lunstad *et al.*, 2015; Kondo *et al.*, 2009; Zhuo and Harrigan, 2023), which is lower than the rates observed in patients suffering from depression. These findings supported our results, indicating that individuals with less favourable SDHs had a higher risk of mortality among individuals with depression compared to those with the most favourable SDHs.

Disadvantaged SDHs have been associated with increased risks of adverse health outcomes among adults with depression, including CVD, cancer and dementia. The association between depression and CVD morbidity and mortality has long been established, with SDHs playing a role in the global burden of CVD (Powell-Wiley *et al.*, 2022). A study found that individuals in the highest quintile of polysocial risk score had nearly four times higher odds of atherosclerotic CVD compared to those in the lowest quintile (Javed *et al.*, 2021). The biological mechanisms linking SDHs to CVD pathogenesis include excess stress hormones, inflammation, immune cell function and cellular ageing (Powell-Wiley *et al.*, 2022). Chronic stress in depression leads to elevated glucocorticoids through activation of the hypothalamic–pituitary–adrenal (HPA) axis, resulting in hyperglycaemia and insulin resistance, which are risk factors for heart diseases (Warriach *et al.*, 2022). In oncology, SDHs impact all aspects of cancer care, from screening to the end of life and survivorship (Tucker-Seeley *et al.*, 2024). Psychiatric disorders were associated with increased risks of cancer incidence (adjusted relative risk, RR = 1.13, 95% CI: 1.06–1.19) and cancer-specific mortality (1.21, 95% CI: 1.16–1.26) (Wang *et al.*, 2020). Social genomic determinants of health, through which contextual factors, particularly one’s neighbourhood, can influence the activity of the cancer genome and the surrounding tumour microenvironment, affecting disease progression and treatment outcomes (Goel *et al.*, 2024).

A systematic review and meta-analysis based on 19 longitudinal cohort studies discovered that a lack of social interaction is associated with incident dementia, including low social participation (RR = 1.41, 95% CI: 1.13–1.75), less frequent social contact (RR = 1.57, 95% CI: 1.32–1.85) and more loneliness (RR = 1.58, 95% CI: 1.19–2.09) (Kuiper *et al.*, 2015). In addition, disadvantaged SDHs, such as low socioeconomic status, education level, food security, and neighbourhood and built environment factors, were associated with a higher incidence of Alzheimer’s disease-related dementia (Majoka and Schimming, 2021). Conversely, higher levels of social engagement have a protective effect on the diagnosis of Alzheimer’s disease-related dementia (Majoka and Schimming, 2021). Furthermore, a prospective study with 64,706 participants showed that individuals with depression have higher risks of dementia compared to those without depression (HR = 1.65, 95% CI: 1.26–2.17) (Yan *et al.*, 2024). These findings were consistent with our findings, and in our study, we focused on the impact of combined SDHs on the morbidity and mortality of adverse health outcomes among adults with depression, which suggested that interventions targeting risk factors strongly associated with disadvantaged SDHs could be beneficial for the health of depression.

Our study found that sociodemographic and behaviour characteristics of individuals also influenced the associations between combined SDHs and all-cause mortality, as well as adverse health outcomes among individuals with depression. Specifically, a meta-analysis demonstrated that among individuals with depression, males had nearly twice the mortality rate compared to females (Cuijpers *et al.*, 2014). Objectively measured physical activity exhibited a positive correlation with socioeconomic status (Stalling *et al.*, 2022). Furthermore, active physical activity exerts a relative protective effect on mortality among depression through enhancing cardiorespiratory fitness, modulating inflammatory processes and promoting beneficial adaptations in homeostatic systems’ response to stress (Belvederi Murri *et al.*, 2018). Consistent with previous studies, lower socioeconomic status was associated with a higher likelihood of current smoking and alcohol consumption, both of which can increase the risk of mortality among individuals with depression (Huang *et al.*, 2023; Moustgaard *et al.*, 2022; Probst *et al.*, 2020; Tam *et al.*, 2020). Notably, our findings revealed that participants without hypertension and diabetes had a higher risk of mortality and incident CVD. This observation may be attributed to individuals with hypertension and diabetes being more attentive to their lifestyle choices and adopting healthier habits (Wakasugi *et al.*, 2022).

This study advances the existing literature by introducing combined SDHs to assess the cumulative burden of social determinants on morbidity and mortality among patients with depression. By consolidating multiple SDHs into a unified metric, our findings provide an empirically validated tool for risk stratification, which may enhance the identification of high-risk subpopulations and inform targeted interventions. Importantly, the consistent associations observed across two independent, nationally representative cohorts underscore the robustness and generalizability of this approach, supporting its potential applicability in diverse clinical and public health settings. Several limitations should be noted in this study. First, owing to data limitations, the combined SDHs were derived from 14 SDHs in the UK Biobank and 9 SDHs in the US NHANES, each capturing different facets and potentially leading to misclassification across populations. Nonetheless, despite the variations in scoring and social contexts, unfavourable combined SDHs were linked to heightened health risks among adults with depression, suggesting the potential generalizability of our findings. Additionally, the combined SDHs should represent a range of factors that influence individuals’ living conditions and overall quality of life, and should be expanded in further studies. Second, the sample size for researching mortality and incidence of cancer and dementia among patients with depression is limited. Nevertheless, this represents our most extensive data collection effort to date. Third, all data regarding SDHs and covariates in this study were gathered at baseline, limiting our ability to capture temporal changes and potentially introducing measurement errors. Future research utilizing repeated measurements would be advantageous. Fourth, while we accounted for the various contributions of SDHs, the scoring system does not adequately capture the interactions among them. There remains a need for a more robust method to evaluate the cumulative impact of SDHs effectively. Fifth, although sensitivity analyses using competing risk models supported the robustness of our findings, residual confounding could remain if unmeasured factors affect CVD and cancer mortality asymmetrically.

## Conclusions

Through the UK Biobank and US NHANES cohorts, we discovered that disadvantaged SDHs were associated with a higher risk of unhealthy outcomes in depression patients. Given that individuals with depression often face more adverse SDHs and that depression can further aggravate these disadvantages, it is essential to prioritize a comprehensive approach to combined SDHs as a core principle in the prevention and management of depression, thereby breaking the cycle of disadvantage.

## Supporting information

10.1017/S2045796025100176.sm001 Qi et al. supplementary material Qi et al. supplementary material

## Data Availability

The UKB data are available through the UK Biobank Access Management System (https://www.ukbiobank.ac.uk/).

## Supplementary material

The supplementary material for this article can be found at https://doi.org/10.1017/S2045796025100176.
