# Supplementary material for: Combined social determinants of health contributed to adverse health outcomes among depression: evidence from two national cohorts
Source: Epidemiol Psychiatr Sci. 2025 Aug 22;34:e43. doi: 10.1017/S2045796025100176 (PMC12450540; doi:10.1017/S2045796025100176)
Supplement: Qi et al. supplementary material [file S2045796025100176sup001.docx]

**Supplementary Material**

**Supplementary Figure 1.** Unadjusted Kaplan-Meier survival curves according to combined SDH group among adults with depression in the UK Biobank and US NHANES cohorts.

**Supplementary Figure 2.** Subgroup analyses of the associations between the combined SDHs with incident CVD, incident cancer and incident dementia among adults with depression in the UK Biobank

**Supplementary table S1.** Assessment of SDHs in the UK Biobank and US NHANES cohorts

**Supplementary table S2.** β coefficients for each SDH and derivation of combined weighted scores in the UK Biobank

**Supplementary table S3.** β coefficients for each SDH and derivation of combined weighted scores in the US NHANES

**Supplementary table S4.** Assessment of covariates

**Supplementary table S5.** Missing values of variables in the UK Biobank and US NHANES cohorts

**Supplementary table S6.** Codes used to identify prevalent comorbidities at baseline

**Supplementary table S7.** Codes used to identify outcomes

**Supplementary table S8.** Baseline characteristics of the study population grouped by combined SDHs in the UK Biobank and US NHANES cohorts

**Supplementary table S9.** Percentages of participants in disadvantaged level for each SDH

**Supplementary table S10.** Associations of the combined SDHs with mortality and incident diseases among adults with depression: excluding participants with CVD and cancer at baseline

**Supplementary table S11.** Associations of the combined SDHs with mortality and incident diseases among adults with depression: excluding participants within two years of follow-up time

**Supplementary table S12.** Associations of the combined SDHs with mortality and incident diseases among adults with depression: using unweighted SDHs score

**Supplementary table S13.** Competing risk analysis using Fine-Gray models in the UK Biobank and US NHANES cohorts

**Supplementary methods** Measurements of covariates


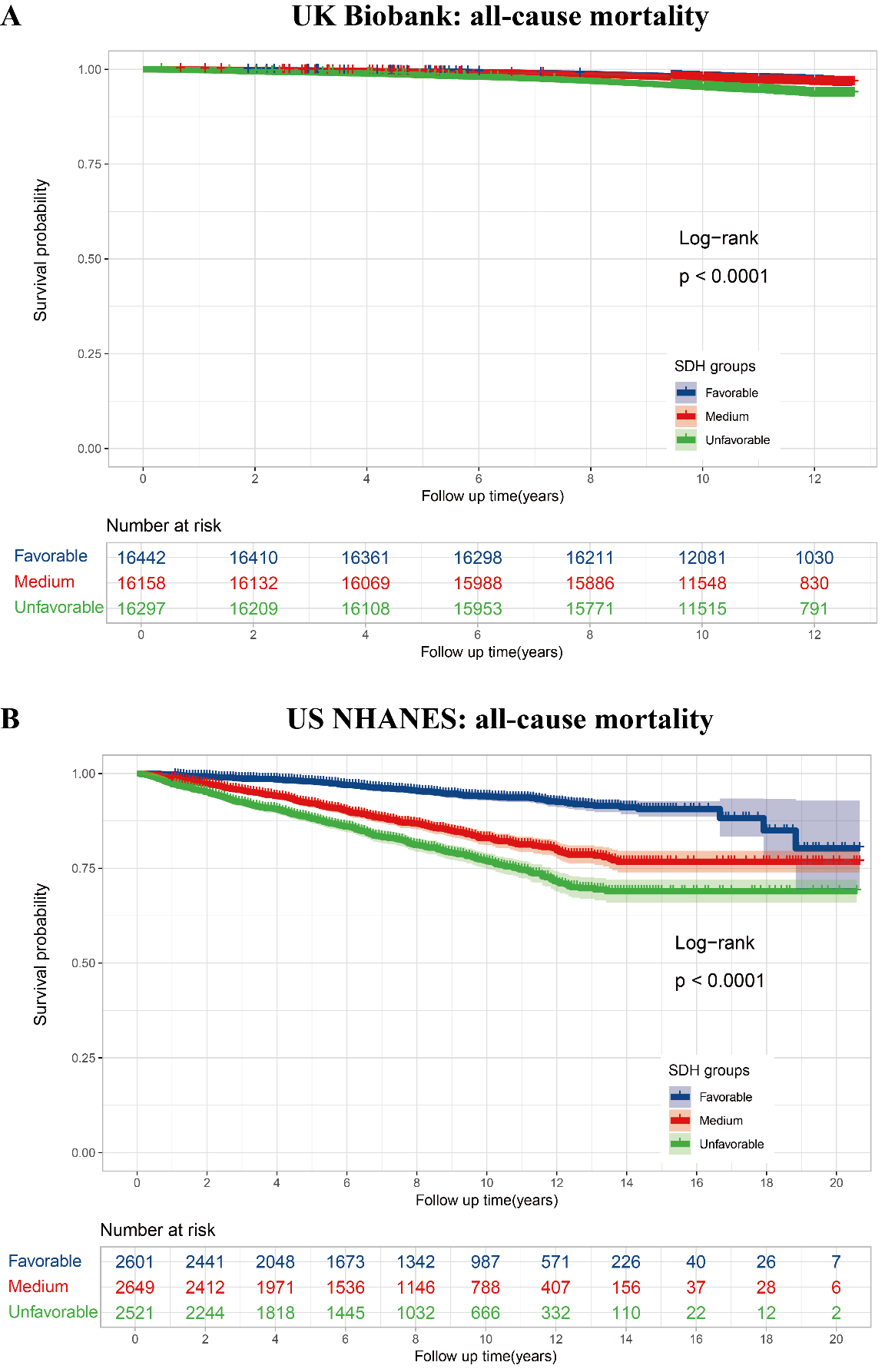


# **Supplementary Figure 1. Unadjusted Kaplan-Meier survival curves according to combined SDH group among adults with depression in the UK Biobank and US NHANES cohorts**

Favorable, medium, and unfavorable represents the bottom, middle, and top tertile of weighted SDH score, respectively. Abbreviations: SDHs, social determinants of health; NHANES, National Health and Nutrition Examination Survey.

**
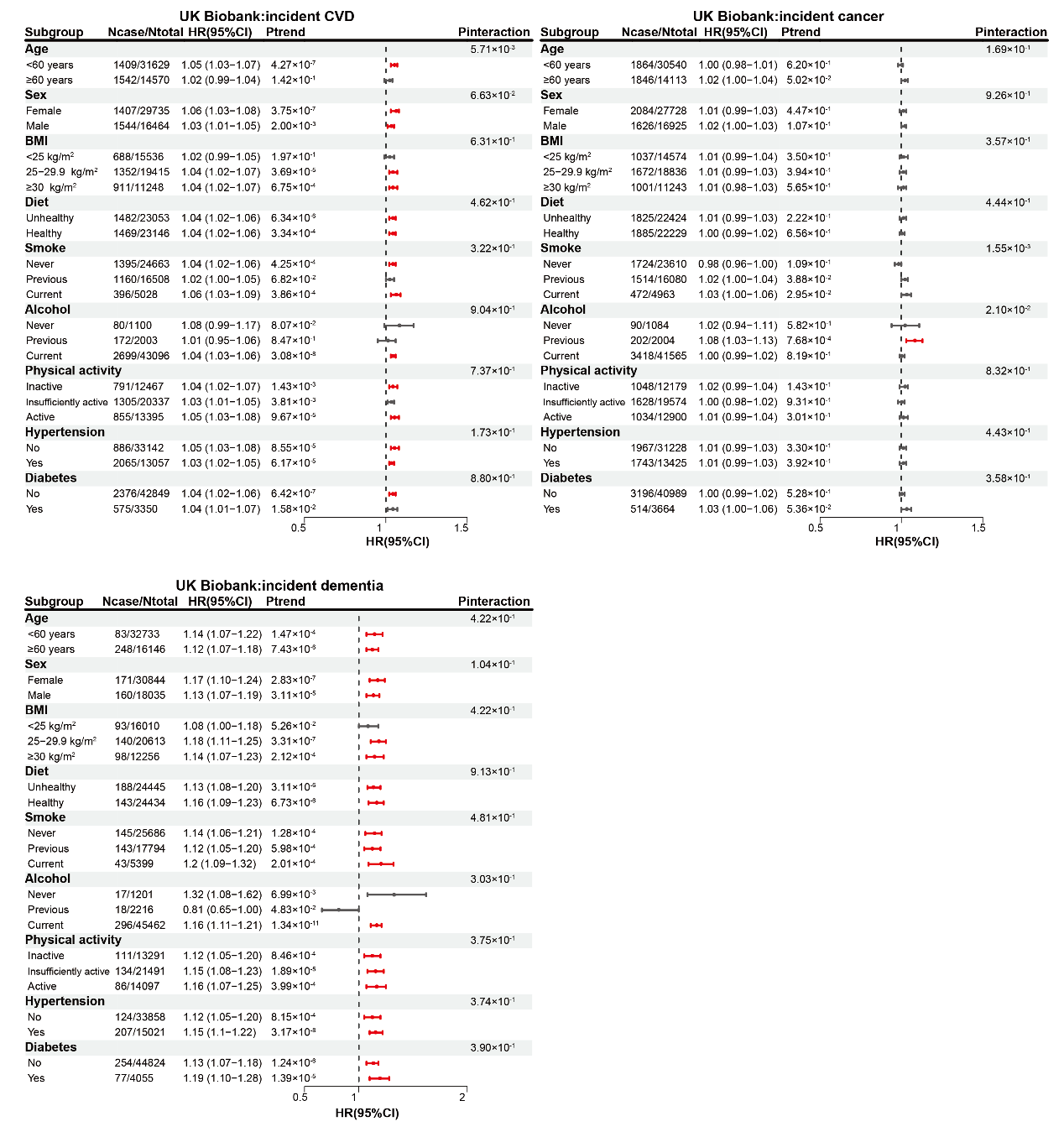
**

# **Supplementary Figure 2. Subgroup analyses of the associations between the combined SDHs with incident CVD, incident cancer and incident dementia among adults with depression in the UK Biobank cohort**

The red points and lines indicate significant results, while the gray represents non-significant findings.

Abbreviations: SDHs, social determinants of health; CVD, cardiovascular disease; CI, confidence interval; HR, hazard ratio.

# Supplementary table S1. Assessment of SDHs in the UK Biobank and US NHANES cohorts

| **Domain** | **Social determinants of health** | **Code*** | **UK biobank** | | **US NHANES** | |
| --- | --- | --- | --- | --- | --- | --- |
|  |  |  | **ID** | **Definition** | **ID** | **Definition** |
| **Financial circumstances** | Household income | 0 | 738 | Average total household income before tax is more than 31,000 | INDFMPIR | family poverty/income ratio≥300% |
|  |  | 1 |  | less than 31,000 |  | family poverty/income ratio<300% |
|  | Employment status | 0 | 6142 | Employed, retired or student | OCQ150, OCD150, OCQ380 | Employed, retired or student |
|  |  | 1 |  | Unemployed |  | Unemployed |
|  | Income quality | 0 | 26411, 26418, 26428 | Below the median of income deprivation score |  | .. |
|  |  | 1 |  | Above the median |  | .. |
|  | Food security | 0 |  | .. | ADFDSEC, FSDAD | Full food security (0 affirmative) |
|  |  | 1 |  | .. |  | Marginal, low, or very low (1-10 affirmative) |
| **Education access and quality** | Education attainment | 0 | 6138 | Highest education level is college or above | DMDEDUC2 | College graduate |
|  |  | 1 |  | Lower than college |  | less than college graduate |
|  | Education quality | 0 | 26414, 26421, 26431 | Below the median of education deprivation score |  | .. |
|  |  | 1 |  | Above the median |  | .. |
| **Health case access and quality** | Health care | 0 | 26413, 26420, 26430 | Below the median of healthcare deprivation score | HUQ030, HUQ040, HUQ041 | Routine place to go for healthcare |
|  |  | 1 |  | Above the median |  | No routine place, or Emergency room/hospital/other |
|  | Health insurance | 0 |  | .. | HID010, HID030A, HIQ011, HIQ031A | Private insurance |
|  |  | 1 |  | .. |  | Government or no insurgence |
| **Neighborhood and built environment** | Accommodation stability | 0 | 680 | Own home outright | HOQ065 | Own home |
|  |  | 1 |  | Rent or other arrangement |  | Rent or other arrangement |
|  | Local crime rate | 0 | 26416, 26434 | Below the median of crime score |  | .. |
|  |  | 1 |  | Above the median |  | .. |
|  | Natural environment | 0 | 24506 | Above the median percentage of home location buffer classed as natural land |  | .. |
|  |  | 1 |  | Below the median |  | .. |
| **Social and community context** | Race/ethnicity | 0 |  | .. | RIDRETH1 | Others |
|  |  | 1 |  | .. |  | Black Race |
|  | Marital status | 0 |  | .. | DMDMARTL | Married or living with a partner |
|  |  | 1 |  | .. |  | Not married nor living with a partner |
|  | Living alone/with partners | 0 | 709 | Living with partners |  | .. |
|  |  | 1 |  | Living alone |  | .. |
|  | Social support | 0 | 2110 | Able to confide in anyone close to you at least once a week |  | .. |
|  |  | 1 |  | less than once a week |  | .. |
|  | Social activity | 0 | 6160 | Attend any group activities once a week or more than |  | .. |
|  |  | 1 |  | Less often than once a week |  | .. |
|  | Social isolation | 0 | 1031 | Visit friend/family or have them visit you once a week or more than |  | .. |
|  |  | 1 |  | less often than once a week |  | .. |
|  | Emotional distress | 0 | 6145 | None |  | .. |
|  |  | 1 |  | Have experienced illness, injury, bereavement, stress within last 2 years |  | .. |

*Code: 1=disadvantaged level; 0= advantaged level.

Abbreviations: SDHs, social determinants of health; NHANES, National Health and Nutrition Examination Survey.

# Supplementary table S2. β coefficients for each SDH and derivation of combined weighted scores in the UK Biobank

| **Social determinants of health** | **Parsimonious Model** | | **Risk score** |
| --- | --- | --- | --- |
|  | **adjusted beta (95%CI)** | **adjusted HR (95%CI)** |  |
| **Financial circumstances** |  |  |  |
| Household income | 0.47 (0.42-0.52) | 1.60 (1.44-1.78) | 1.73 |
| Employment status | 0.82 (0.75-0.89) | 2.27 (1.99-2.59) | 3.02 |
| Income quality | 0.16 (0.11-0.21) | 1.17 (1.06-1.29) | 0.59 |
| **Education access and quality** | |  |  |
| Education attainment | 0.11 (0.06-0.16) | 1.11 (1.01-1.23) | 0.41 |
| Education quality | 0.21 (0.16-0.26) | 1.23 (1.12-1.36) | 0.77 |
| **Health case access and quality** | |  |  |
| Health care | 0.26 (0.21-0.31) | 1.30 (1.18-1.43) | 0.96 |
| **Neighborhood and built environment** | |  |  |
| Accommodation stability | 0.65 (0.58-0.72) | 1.91 (1.67-2.17) | 2.39 |
| Local crime rate | 0.03 (-0.02-0.08) | 1.03 (0.93-1.13) | 0.11 |
| Natural environment | 0.14 (0.09-0.19) | 1.15 (1.05-1.27) | 0.52 |
| **Social and community context** | |  |  |
| Living alone/with partners | 0.32 (0.27-0.37) | 1.37 (1.23-1.52) | 1.18 |
| Social support | 0.14 (0.09-0.19) | 1.15 (1.04-1.27) | 0.52 |
| Social isolation | 0.10 (0.04-0.16) | 1.11 (0.99-1.24) | 0.37 |
| Social activity | 0.07 (0.02-0.12) | 1.07 (0.97-1.19) | 0.26 |
| Emotional distress | 0.32 (0.27-0.37) | 1.38 (1.25-1.52) | 1.18 |
| ***Weighted score (total): 14*** | |  |  |

*Adjusted for Age, sex, BM, smoking status, drinking status, diet, physical activity, prevalence of hypertension and diabetes.

Abbreviations: SDHs, social determinants of health; HR, hazard ratio; CI, confidence interval.

# Supplementary table S3. β coefficients for each SDH and derivation of combined weighted scores in the US NHANES

| **Social determinants of health** | **Parsimonious Model** | | **Risk score** |
| --- | --- | --- | --- |
|  | **adjusted beta (95%CI)** | **adjusted HR (95%CI)** |  |
| **Financial circumstances** | |  |  |
| Household income | 0.35 (0.26-0.44) | 1.41 (1.19-1.68) | 1.41 |
| Employment status | 0.72 (0.62-0.82) | 2.05 (1.68-2.51) | 2.91 |
| Food security | 0.12 (0.05-0.19) | 1.13 (0.99-1.30) | 0.48 |
| **Education access and quality** | |  |  |
| Education attainment | 0.27 (0.15-0.39) | 1.30 (1.04-1.64) | 1.09 |
| **Health case access and quality** | |  |  |
| Health care | 0.02 (-0.08-0.12) | 1.02 (0.84-1.23) | 0.08 |
| Health insurance | 0.15 (0.08-0.22) | 1.16 (1.02-1.33) | 0.61 |
| **Neighborhood and built environment** | |  |  |
| Accommodation stability | 0.14 (0.07-0.21) | 1.15 (1.00-1.32) | 0.57 |
| **Social and community context** | |  |  |
| Race/ethnicity | 0.13 (0.05-0.21) | 1.14 (0.97-1.33) | 0.52 |
| Marital status | 0.33 (0.26-0.4) | 1.40 (1.22-1.60) | 1.33 |
| ***Weighted score (total): 9*** | |  |  |

*Adjusted for Age, sex, BM, smoking status, drinking status, diet, physical activity, prevalence of hypertension and diabetes.

Abbreviations: SDHs, social determinants of health; HR, hazard ratio; CI, confidence interval.

# Supplementary table S4. Assessment of covariates

| **Covariates** | **UK Biobank** | **NHANES** |
| --- | --- | --- |
| Age | 21003 | RIDAGEYR |
| Sex | 31 | RIAGENDR |
| Body mass index | 21001 | BMXBMI |
| Smoking status | 20116 | SMQ020, SMQ040 |
| Drinking status | 20117 | ALQ100, ALD100, ALQ101, ALQ111, ALQ120Q, ALQ121 |
| Physical activity | 22033 | PAD200, PAD320, PADMETS, PAQ605, PAQ610, PAQ620, PAQ625 |
| Hypertension | 41270 and 41280, ICD I10 to I15 | BPQ020, BPQ040A, BPXSY1, BPXDI1 |
| Diabetes | 41270 and 41280, ICD E10 to E14 | LBXGH, DIQ010, DIQ175W, DIQ050, DIQ070, DID070 |
| Diet | Dietary diversity score* | HEI2010, HEI2015 |

Abbreviations: ICD, International Classification of Diseases; NHANES, National Health and Nutrition Examination Survey.

*Dietary diversity score was obtained from 24-hour dietary recalls and based on a dietary recommendation according to a previous study [1]. Five items of the recommendations were included, including grain products (whole grains and non-whole grains), Vegetables (dark green leafy, vitamin A-rich, starchy tubers, and other), Fruits (citrus, vitamin A-rich and other), Meat and protein alternatives (red meat, poultry, fish and seafood, organ meat, eggs and legumes, and nuts), and Dairy products (milk, yoghurt and cheese). We calculated the average value of repeated diet evaluations. For each large category, if a participant consumes any food in this category, the score will increase. However, in the same small group, different diverse food intakes will not be repeatedly calculated. Therefore, the dietary diversity score ranged from 0 to 18.

# Supplementary table S5. Missing values of variables in the UK Biobank and US NHANES cohorts

| **Covariates** | **UK Biobank** | | **US NHANES** | |
| --- | --- | --- | --- | --- |
|  | Missing number | Missing percentage | Missing number | Missing percentage |
| Body mass index | 1,250 | 2.56% | 91 | 1.17% |
| Smoking status | 72 | 0.14% | 2 | 0.03% |
| Drinking status | 32 | 0.07% | 22 | 0.28% |
| Physical activity | 5,710 | 11.68% | 152 | 1.96% |
| Diabetes | 0 | 0% | 9 | 0.12% |
| Diet | 17,426 | 35.63% | 1,159 | 14.91% |

Abbreviations: NHANES, National Health and Nutrition Examination Survey.

# Supplementary table S6. Codes used to identify prevalent comorbidities at baseline

| **Prevalent diseases** | **UK Biobank** | | **US NHANES** |
| --- | --- | --- | --- |
|  | **ICD-10** | **Self-reported** | **Self-reported** |
| CVD | I20-I25, I60-64 | 6150 (1,2,3)  20002(1074,1075,1081,1583,1086,1491) | MCQ160B, MCQ160C, MCQ160D, MCQ160E, MCQ160F |
| Cancer | C00-C97 | 20001 | MCQ220 |
| Dementia | A810, F00, F01, F02, F03, F051, F106, G30, G310, G311, G318, I673 | 20003 (Prescriptions including memantine, donepezil, galantamine and rivastigmine) | .. |

Abbreviations: CVD, cardiovascular disease; ICD, International Classification of Diseases; NHANES, National Health and Nutrition Examination Survey.

# Supplementary table S7. Codes used to identify outcomes

|  | **UK Biobank (ICD-10)** |
| --- | --- |
| **Mortality** |  |
| All-cause mortality | ·· |
| CVD mortality | I00-I99 |
| Cancer mortality | C00-C97 |
| **Incident non-fatal outcomes** | |
| Incident CVD | I20-I25, I60-I64 |
| Cancer | C00-C97* |
| Dementia | A810, F00, F01, F02, F03, F051, F106, G30, G310, G311, G318, I673 |

Abbreviations: CVD, cardiovascular disease; ICD, International Classification of Diseases

* Except for non-melanoma skin cancer (ICD-10 code, C44).

# Supplementary table S8. Baseline characteristics of the study population grouped by combined SDHs in the UK Biobank and US NHANES cohort

|  | **UK Biobank** | | | | | **US NHANES** | | | | |
| --- | --- | --- | --- | --- | --- | --- | --- | --- | --- | --- |
|  | Total  (n = 48,897) | Favorable  (n = 16,442) | Medium  (n = 16,158) | Unfavorable (n = 16,297) | P | Total  (n = 7,771) | Favorable  (n = 2,601) | Medium  (n = 2,649) | Unfavorable  (n = 2,521) | P |
| Weighted SDH score range |  | [0–2.84] | (2.84–4.91] | (4.91–14] |  |  | [0–4.2] | (4.2–6.6] | (6.6–10.0] |  |
| Sex, n (%) |  |  |  |  | <2.00E-16 |  |  |  |  | 1.25E-07 |
| Female | 30,850 (63.1) | 9,828 (59.8) | 10,478 (64.8) | 10,544 (64.7) |  | 4,696 (60.4) | 1,484 (57.1) | 1,582 (59.7) | 1,630 (64.7) |  |
| Male | 18,047 (36.9) | 6,614 (40.2) | 5,680 (35.2) | 5,753 (35.3) |  | 3,075 (39.6) | 1,117 (42.9) | 1,067 (40.3) | 891 (35.3) |  |
| Age (years), Median (Q1,Q3) | 55 (49, 61) | 55 (49, 61) | 56 (49, 62) | 55 (48, 61) | 2.66E-10 | 48 (34, 62) | 43 (32, 55) | 50 (34, 64) | 54 (38, 66) | <2.00E-16 |
| BMI (kg/m^2^), Median (Q1,Q3) | 26.9 (24.1, 30) | 26.2 (23.8, 29.1) | 26.9 (24.1, 29.9) | 27.4 (24.5, 31) | <2.00E-16 | 29.3 (24.9, 34.6) | 28.7 (24.6, 33.9) | 29.5 (25, 34.8) | 30 (25.2, 35.1) | 3.20E-05 |
| Smoke, n (%) |  |  |  |  | <2.00E-16 |  |  |  |  | <2.00E-16 |
| Never | 25,693 (52.5) | 9,333 (56.8) | 8,720 (54.0) | 7,640 (46.9) |  | 3,574 (46.0) | 1,399 (53.8) | 1,191 (45.0) | 984 (39.0) |  |
| Previous | 17,802 (36.4) | 5,952 (36.2) | 6,033 (37.3) | 5,817 (35.7) |  | 1,831 (23.6) | 613 (23.6) | 656 (24.8) | 562 (22.3) |  |
| Current | 5,402 (11.0) | 1,157 (7.0) | 1,405 (8.7) | 2,840 (17.4) |  | 2,366 (30.4) | 589 (22.6) | 802 (30.3) | 975 (38.7) |  |
| Alcohol, n (%) |  |  |  |  | <2.00E-16 |  |  |  |  | <2.00E-16 |
| Never | 1,202 (2.5) | 252 (1.5) | 403 (2.5) | 547 (3.4) |  | 1,943 (25.0) | 549 (21.1) | 675 (25.5) | 719 (28.5) |  |
| Previous | 2,216 (4.5) | 466 (2.8) | 610 (3.8) | 1140 (7.0) |  | 1,208 (15.5) | 267 (10.3) | 411 (15.5) | 530 (21.0) |  |
| Current | 45,479 (93.0) | 15,724 (95.6) | 15,145 (93.7) | 14,610 (89.6) |  | 4,620 (59.5) | 1,785 (68.6) | 1,563 (59) | 1,272 (50.5) |  |
| Physical activity, n (%) |  |  |  |  | 7.78E-01 |  |  |  |  | <2.00E-16 |
| Inactive | 13,298 (27.2) | 4,463 (27.1) | 4,346 (26.9) | 4,489 (27.5) |  | 4,823 (62.1) | 1,464 (56.3) | 1,578 (59.6) | 1,781 (70.6) |  |
| Insufficiently active | 21,497 (44.0) | 7,231 (44.0) | 7,138 (44.2) | 7,128 (43.7) |  | 1,226 (15.8) | 406 (15.6) | 414 (15.6) | 406 (16.1) |  |
| Active | 14,102 (28.8) | 4,748 (28.9) | 4,674 (28.9) | 4,680 (28.7) |  | 1,722 (22.2) | 731 (28.1) | 657 (24.8) | 334 (13.2) |  |
| Diet, n (%) |  |  |  |  | <2.00E-16 |  |  |  |  | 1.22E-02 |
| Unhealthy | 24,450 (50.0) | 7,822 (47.6) | 7,943 (49.2) | 8,685 (53.3) |  | 3,886 (50.0) | 1,239 (47.6) | 1,354 (51.1) | 1,293 (51.3) |  |
| Healthy | 24,447 (50.0) | 8,620 (52.4) | 8,215 (50.8) | 7,612 (46.7) |  | 3,885 (50.0) | 1,362 (52.4) | 1,295 (48.9) | 1,228 (48.7) |  |
| Hypertension, n (%) |  |  |  |  | <2.00E-16 |  |  |  |  | <2.00E-16 |
| No | 33,863 (69.3) | 12,197 (74.2) | 11,200 (69.3) | 10,466 (64.2) |  | 3,996 (51.4) | 1,610 (61.9) | 1,385 (52.3) | 1,001 (39.7) |  |
| Yes | 15,034 (30.7) | 4,245 (25.8) | 4,958 (30.7) | 5,831 (35.8) |  | 3,775 (48.6) | 991 (38.1) | 1,264 (47.7) | 1,520 (60.3) |  |
| Diabetes, n (%) |  |  |  |  | <2.00E-16 |  |  |  |  | <2.00E-16 |
| No | 44,837 (91.7) | 15,576 (94.7) | 14,969 (92.6) | 14,292 (87.7) |  | 6,167 (79.4) | 2,245 (86.3) | 2,083 (78.6) | 1,839 (72.9) |  |
| Yes | 4,060 (8.3) | 866 (5.3) | 1,189 (7.4) | 2,005 (12.3) |  | 1,604 (20.6) | 356 (13.7) | 566 (21.4) | 682 (27.1) |  |

Abbreviations: SDH, social determinant of health; NHANES, National Health and Nutrition Examination Survey.

For continuous variables, differences across three groups were tested though Kruskal-Wallis K tests when data were not normally distributed or homogeneity of variance. For categorical variables, differences across groups were tested by the χ^2^ test.

# Supplementary table S9. Percentages of participants in disadvantaged level for each SDH*

| **Social determinants of health*** | **UK Biobank** | | | | **US NHANES** | | | |
| --- | --- | --- | --- | --- | --- | --- | --- | --- |
|  | Total  (n = 48,897) | Favorable  (n = 16,442) | Medium  (n = 16,158) | Unfavorable (n = 16,297) | Total  (n = 7,771) | Favorable  (n = 2,601) | Medium  (n = 2,649) | Unfavorable  (n = 2,521) |
| Weighted SDH score range |  | [0–2.84] | (2.84–4.91] | (4.91–14] |  | [0–4.2] | (4.2–6.6] | (6.6–10.0] |
| ***Financial circumstances*** |  |  |  |  |  |  |  |  |
| Low household income | 22,107 (45.2) | 1,471 (8.9) | 6,806 (42.1) | 13,830 (84.9) | 5,835 (75.1) | 1,120 (43.1) | 2,217 (83.7) | 2,498 (99.1) |
| Unemployed | 4,934 (10.1) | 0 (0) | 638 (3.9) | 4,296 (26.4) | 4,311 (55.5) | 273 (10.5) | 1,517 (57.3) | 2,521 (100) |
| Lower income quality | 25,206 (51.5) | 3,142 (19.1) | 8,939 (55.3) | 13,125 (80.5) | .. | .. | .. | .. |
| Food insecurity | .. | .. | .. | .. | 3,578 (46.0) | 685 (26.3) | 1,263 (47.7) | 1,630 (64.7) |
| ***Education access and quality*** |  | |  |  |  |  |  |  |
| Lower education attainment | 28,460 (58.2) | 7,139 (43.4) | 9,434 (58.4) | 11,887 (72.9) | 6,621 (85.2) | 1,806 (69.4) | 2,384 (90.0) | 2,431 (96.4) |
| Lower education quality | 24,464 (50.0) | 2,966 (18.0) | 8,839 (54.7) | 12,659 (77.7) | .. | .. | .. | .. |
| ***Health case access and quality*** |  | |  |  |  |  |  |  |
| Difficulty in access to health care | 24,539 (50.2) | 3,047 (18.5) | 8,748 (54.1) | 12,744 (78.2) | 1,774 (22.8) | 470 (18.1) | 713 (26.9) | 591 (23.4) |
| Lack of health insurance | .. | .. | .. | .. | 4,611 (59.3) | 751 (28.9) | 1,685 (63.6) | 2,175 (86.3) |
| ***Neighborhood and built environment*** |  | |  |  |  |  |  |  |
| Accommodation instability | 4,791 (9.8) | 18 (0.1) | 310 (1.9) | 4,463 (27.4) | 3,600 (46.3) | 777 (29.9) | 1,133 (42.8) | 1,690 (67.0) |
| Higher local crime rate | 24,469 (50.0) | 5,198 (31.6) | 8,224 (50.9) | 11,047 (67.8) | .. | .. | .. | .. |
| Lack of natural environment | 24,441 (50.0) | 5,941 (36.1) | 8,150 (50.4) | 10,350 (63.5) | .. | .. | .. | .. |
| ***Social and community context*** |  | |  |  |  |  |  |  |
| Black race | .. | .. | .. | .. | 1,676 (21.6) | 385 (14.8) | 549 (20.7) | 742 (29.4) |
| Unmarried | .. | .. | .. | .. | 3,856 (49.6) | 611 (23.5) | 1,267 (47.8) | 1,978 (78.5) |
| Living alone | 10,822 (22.1) | 724 (4.4) | 2,910 (18.0) | 7,188 (44.1) | .. | .. | .. | .. |
| Lack of social support | 13,051 (26.7) | 2,990 (18.2) | 4,106 (25.4) | 5,955 (36.5) | .. | .. | .. | .. |
| Social inactive | 15,124 (30.9) | 4,110 (25.0) | 5,029 (31.1) | 5,985 (36.7) | .. | .. | .. | .. |
| Social isolation | 11,376 (23.3) | 3,812 (23.2) | 3,582 (22.2) | 3,982 (24.4) | .. | .. | .. | .. |
| Emotional distress | 25,226 (51.6) | 5,027 (30.6) | 8,594 (53.2) | 11,605 (71.2) | .. | .. | .. | .. |

* Data are presented as n (row %).

Abbreviations: SDH, social determinant of health; NHANES, National Health and Nutrition Examination Survey.

# Supplementary table S10. Associations of the combined SDHs with mortality and incident diseases among adults with depression: excluding participants with CVD and cancer at baseline

|  | **UK Biobank cohort** | | | | **US NHANES cohort** | | | |
| --- | --- | --- | --- | --- | --- | --- | --- | --- |
|  | **Favorable** | **Medium** | **Unfavorable** | **P trend** | **Favorable** | **Medium** | **Unfavorable** | **P trend** |
| **Weighted SDH score range** | [0–2.84] | (2.84–4.91] | (4.91–14] |  | [0–4.2] | (4.2–6.6] | (6.6–10.0] |  |
| **All-cause mortality** |  |  |  |  |  |  |  |  |
| Number of participants | 14,460 | 14,058 | 13,565 |  | 2,203 | 1,810 | 1,500 |  |
| Number of cases; person-years | 307; 154,619 | 300; 147,980 | 531;142,026 |  | 77; 188,884 | 145; 14,968 | 207; 12,897 |  |
| HR (95% CI) in Model 1 | 1 (ref) | 1.03 (0.88-1.21) | 2.03 (1.76-2.34) | <2E-16 | 1 (ref) | 1.85 (1.40–2.46) | 2.87 (2.20–3.76) | 2.54E-15 |
| HR (95% CI) in Model 2 | 1 (ref) | 0.98 (0.83-1.14) | 1.71 (1.48-1.98) | 1.42E-14 | 1 (ref) | 1.65 (1.25–2.19) | 2.29 (1.74–3.02) | 2.06E-09 |
| **Cardiovascular disease mortality** |  |  |  |  |  |  |  |  |
| Number of cases | 29 | 39 | 72 |  | 14 | 43 | 49 |  |
| HR (95% CI) in Model 1 | 1 (ref) | 1.45 (0.89-2.34) | 3.04 (1.98-4.69) | 1.13E-07 | 1 (ref) | 2.63 (1.43–4.87) | 3.16 (1.72–5.82) | 3.07E-04 |
| HR (95% CI) in Model 2 | 1 (ref) | 1.37 (0.84-2.21) | 2.58 (1.66-4.00) | 8.86E-06 | 1 (ref) | 2.31 (1.25–4.29) | 2.39 (1.28–4.45) | 1.30E-02 |
| **Cancer mortality** |  |  |  |  |  |  |  |  |
| Number of cases | 96 | 102 | 144 |  | 24 | 24 | 34 |  |
| HR (95% CI) in Model 1 | 1 (ref) | 1.08 (0.82-1.43) | 1.73 (1.33-2.24) | 2.45E-05 | 1 (ref) | 0.94 (0.53–1.68) | 1.49 (0.87–2.56) | 1.28E-01 |
| HR (95% CI) in Model 2 | 1 (ref) | 1.03 (0.78-1.37) | 1.42 (1.09-1.85) | 7.50E-03 | 1 (ref) | 0.83 (0.47–1.49) | 1.26 (0.72–2.20) | 3.71E-01 |
| **Incident cardiovascular disease** |  | |  |  |  |  |  |  |
| Number of participants | 14,640 | 14,058 | 13,565 |  |  |  |  |  |
| Number of cases; person-years | 777; 151,034 | 912;143969 | 962;137,741 |  |  |  |  |  |
| HR (95% CI) in Model 1 | 1 (ref) | 1.24 (1.13-1.37) | 1.45 (1.32-1.59) | 1.72E-14 | .. | .. | .. | .. |
| HR (95% CI) in Model 2 | 1 (ref) | 1.13 (1.03-1.24) | 1.16 (1.05-1.28) | 2.76E-03 | .. | .. | .. | .. |
| **Incident cancer** |  |  |  |  |  |  |  |  |
| Number of participants | 14,640 | 14,058 | 13,565 |  |  |  |  |  |
| Number of cases; person-years | 1,152;149,773 | 1,138;143,382 | 1,129; 137,729 |  |  |  |  |  |
| HR (95% CI) in Model 1 | 1 (ref) | 1.01 (0.93-1.10) | 1.09 (1.01-1.19) | 3.72E-02 | .. | .. | .. | .. |
| HR (95% CI) in Model 2 | 1 (ref) | 0.98 (0.91-1.07) | 1.01 (0.93-1.10) | 8.45E-01 | .. | .. | .. | .. |
| **Incident dementia** |  |  |  |  |  |  |  |  |
| Number of participants | 14,638 | 14,054 | 13,560 |  |  |  |  |  |
| Number of cases; person-years | 45; 154,497 | 80;147,773 | 89; 141,763 |  |  |  |  |  |
| HR (95% CI) in Model 1 | 1 (ref) | 1.73 (1.20-2.50) | 2.22 (1.55-3.18) | 1.34E-05 | .. | .. | .. | .. |
| HR (95% CI) in Model 2 | 1 (ref) | 1.68 (1.16-2.42) | 2.03 (1.41-2.92) | 1.73E-04 | .. | .. | .. | .. |

Note: Model 1 was adjusted for age and sex; Model 2 was additionally adjusted for BMI, smoking status, drinking status, diet, physical activity, prevalence of hypertension and diabetes.

Abbreviations: SDH, social determinant of health; NHANES, National Health and Nutrition Examination Survey.

# Supplementary table S11. Associations of the combined SDHs with mortality and incident diseases among adults with depression: excluding participants within two years of follow-up time

|  | **UK Biobank cohort** | | | | **US NHANES cohort** | | | |
| --- | --- | --- | --- | --- | --- | --- | --- | --- |
|  | **Favorable** | **Medium** | **Unfavorable** | **Ptrend** | **Favorable** | **Medium** | **Unfavorable** | **Ptrend** |
| **Weighted SDH score range** | [0–2.84] | (2.84–4.91] | (4.91–14] |  | [0–4.2] | (4.2–6.6] | (6.6–10.0] |  |
| **All-cause mortality** |  |  |  |  |  |  |  |  |
| Number of participants | 16,410 | 16,132 | 16,209 |  | 2,325 | 2,117 | 1,885 |  |
| Number of cases; person-years | 377; 173,188 | 414; 169,552 | 752; 169,498 |  | 109; 21,002 | 275; 19,154 | 345; 12,897 |  |
| HR (95% CI) in Model 1 | 1 (ref) | 1.11 (0.96-1.27) | 2.15 (1.90-2.44) | <2E-16 | 1 (ref) | 1.77 (1.41–2.22) | 2.55 (2.04–3.18) | <2E-16 |
| HR (95% CI) in Model 2 | 1 (ref) | 1.04 (0.90-1.19) | 1.75 (1.54-1.99) | <2E-16 | 1 (ref) | 1.55 (1.23–1.95) | 1.98 (1.57–2.48) | 2.68E-09 |
| **Cardiovascular disease mortality** |  |  |  |  |  |  |  |  |
| Number of cases | 34 | 46 | 108 |  | 26 | 78 | 88 |  |
| HR (95% CI) in Model 1 | 1 (ref) | 1.40 (0.90-2.18) | 3.56 (2.42-5.24) | 2.57E-12 | 1 (ref) | 1.81 (1.15–2.85) | 2.39 (1.52–3.74) | 1.23E-04 |
| HR (95% CI) in Model 2 | 1 (ref) | 1.28 (0.82-1.99) | 2.74 (1.85-4.07) | 4.75E-08 | 1 (ref) | 1.58 (1.00–2.50) | 1.81 (1.14–2.87) | 1.65E-02 |
| **Cancer mortality** |  |  |  |  |  |  |  |  |
| Number of cases | 125 | 139 | 209 |  | 33 | 58 | 65 |  |
| HR (95% CI) in Model 1 | 1 (ref) | 1.09 (0.85-1.38) | 1.74 (1.40-2.18) | 3.59E-07 | 1 (ref) | 1.26 (0.81–1.95) | 1.64 (1.07–2.53) | 2.02E-02 |
| HR (95% CI) in Model 2 | 1 (ref) | 1.03 (0.81-1.31) | 1.46 (1.16-1.83) | 6.66E-04 | 1 (ref) | 1.11 (0.72–1.73) | 1.36 (0.87–2.13) | 1.50E-01 |
| **Incident cardiovascular disease** |  |  |  |  |  |  |  |  |
| Number of participants | 15,719 | 15,203 | 14,757 |  |  |  |  |  |
| Number of cases; person-years | 731; 163,242 | 871; 156,864 | 946; 151,185 |  |  |  |  |  |
| HR (95% CI) in Model 1 | 1 (ref) | 1.26 (1.14-1.39) | 1.50 (1.36-1.65) | <2E-16 | .. | .. | .. | .. |
| HR (95% CI) in Model 2 | 1 (ref) | 1.14 (1.03-1.26) | 1.22 (1.10-1.35) | 9.35E-05 | .. | .. | .. | .. |
| **Incident cancer** |  |  |  |  |  |  |  |  |
| Number of participants | 14,968 | 14,601 | 14,531 |  |  |  |  |  |
| Number of cases; person-years | 1,059; 154,524 | 1,052; 150,278 | 1,117; 148,867 |  |  |  |  |  |
| HR (95% CI) in Model 1 | 1 (ref) | 1.00 (0.92-1.09) | 1.11 (1.02-1.21) | 1.24E-02 | .. | .. | .. | .. |
| HR (95% CI) in Model 2 | 1 (ref) | 0.96 (0.88-1.05) | 1.00 (0.92-1.09) | 9.47E-01 | .. | .. | .. | .. |
| **Incident dementia** |  |  |  |  |  |  |  |  |
| Number of participants | 16,405 | 16,126 | 16,190 |  |  |  |  |  |
| Number of cases; person-years | 64; 172,988 | 109; 169,285 | 146; 168,973 |  |  |  |  |  |
| HR (95% CI) in Model 1 | 1 (ref) | 1.61 (1.18-2.19) | 2.39 (1.78-3.21) | 2.72E-09 | .. | .. | .. | .. |
| HR (95% CI) in Model 2 | 1 (ref) | 1.51 (1.10-2.05) | 2.01 (1.49-2.72) | 3.75E-06 | .. | .. | .. | .. |

Note: Model 1 was adjusted for age and sex; Model 2 was additionally adjusted for BMI, smoking status, drinking status, diet, physical activity, prevalence of hypertension and diabetes.

Abbreviations: SDH, social determinant of health; NHANES, National Health and Nutrition Examination Survey.

# Supplementary table S12. Associations of the combined SDHs with mortality and incident diseases among adults with depression: using unweighted SDHs score

|  | **UK Biobank cohort** | | | | **US NHANES cohort** | | | |
| --- | --- | --- | --- | --- | --- | --- | --- | --- |
|  | **Favorable** | **Medium** | **Unfavorable** | **Ptrend** | **Favorable** | **Medium** | **Unfavorable** | **Ptrend** |
| **Unweighted SDH score range** | [0–3] | [4–6] | [7–14] |  | [0–3] | [4–5] | [6–9] |  |
| **All-cause mortality** |  |  |  |  |  |  |  |  |
| Number of participants | 13,454 | 19,798 | 15,645 |  | 2,285 | 2,548 | 2,938 |  |
| Number of cases; person-years | 359;142,001 | 576; 207,535 | 748;162,869 |  | 211;18,479 | 363; 18,968 | 369; 21,132 |  |
| HR (95% CI) in Model 1 | 1 (ref) | 1.13 (0.99-1.29) | 2.06 (1.81-2.34) | <2E-16 | 1 (ref) | 1.60 (1.35–1.90) | 2.55 (2.04–3.18) | 2.55E-16 |
| HR (95% CI) in Model 2 | 1 (ref) | 1.04 (0.91-1.19) | 1.63 (1.43-1.86) | 6.87E-16 | 1 (ref) | 1.39 (1.17–1.65) | 1.59 (1.33–1.89) | 3.76E-07 |
| **Cardiovascular disease mortality** |  |  |  |  |  |  |  |  |
| Number of cases | 30 | 71 | 114 |  | 54 | 103 | 96 |  |
| HR (95% CI) in Model 1 | 1 (ref) | 1.69 (1.10-2.58) | 3.83 (2.56-5.73) | 3.52E-13 | 1 (ref) | 1.71 (1.23–2.38) | 2.15 (1.54–3.01) | 7.83E-06 |
| HR (95% CI) in Model 2 | 1 (ref) | 1.52 (0.99-2.34) | 2.87 (1.90-4.33) | 2.89E-08 | 1 (ref) | 1.44 (1.03–2.02) | 1.64 (1.16–2.32) | 5.87E-03 |
| **Cancer mortality** |  |  |  |  |  |  |  |  |
| Number of cases | 121 | 209 | 213 |  | 56 | 81 | 71 |  |
| HR (95% CI) in Model 1 | 1 (ref) | 1.20 (0.96-1.5) | 1.73 (1.38-2.16) | 7.20E-07 | 1 (ref) | 1.35 (0.96–1.90) | 1.48 (1.04–2.11) | 2.88E-02 |
| HR (95% CI) in Model 2 | 1 (ref) | 1.12 (0.90-1.4) | 1.41 (1.12-1.77) | 0.002462 | 1 (ref) | 1.22 (0.86–1.72) | 1.23 (0.85–1.77) | 2.86E-01 |
| **Incident cardiovascular disease** |  |  |  |  |  |  |  |  |
| Number of participants | 12,954 | 18,850 | 14,395 |  |  |  |  |  |
| Number of cases; person-years | 723; 16,3415 | 1,199; 157,035 | 1,029; 157,035 |  |  |  |  |  |
| HR (95% CI) in Model 1 | 1 (ref) | 1.19 (1.08-1.30) | 1.48 (1.34-1.63) | 5.13E-16 | .. | .. | .. | .. |
| HR (95% CI) in Model 2 | 1 (ref) | 1.09 (0.99-1.19) | 1.18 (1.07-1.30) | 8.06E-04 | .. | .. | .. | .. |
| **Incident cancer** |  |  |  |  |  |  |  |  |
| Number of participants | 12,329 | 18,044 | 14,280 |  |  |  |  |  |
| Number of cases; person-years | 1,000; 154,703 | 1,522; 15,0461 | 1,188; 149,094 |  |  |  |  |  |
| HR (95% CI) in Model 1 | 1 (ref) | 1.06 (0.98-1.15) | 1.12 (1.03-1.22) | 6.79E-03 | .. | .. | .. | .. |
| HR (95% CI) in Model 2 | 1 (ref) | 1.02 (0.94-1.11) | 1.01 (0.92-1.10) | 8.85E-01 | .. | .. | .. | .. |
| **Incident dementia** |  |  |  |  |  |  |  |  |
| Number of participants | 13,447 | 19,796 | 15,636 |  |  |  |  |  |
| Number of cases; person-years | 59; 173,026 | 130; 169,316 | 142; 169,086 |  |  |  |  |  |
| HR (95% CI) in Model 1 | 1 (ref) | 1.54 (1.13-2.09) | 2.53 (1.87-3.43) | 3.90E-10 | .. | .. | .. | .. |
| HR (95% CI) in Model 2 | 1 (ref) | 1.42 (1.04-1.93) | 2.06 (1.51-2.81) | 2.22E-06 | .. | .. | .. | .. |

Note: Model 1 was adjusted for age and sex; Model 2 was additionally adjusted for BMI, smoking status, drinking status, diet, physical activity, prevalence of hypertension and diabetes.

Abbreviations: SDH, social determinant of health; NHANES, National Health and Nutrition Examination Survey.

# Supplementary table S13. Competing risk analysis using Fine-Gray models in the UK Biobank and US NHANES cohorts

|  | **UK Biobank cohort** | | | | **US NHANES cohort** | | | |
| --- | --- | --- | --- | --- | --- | --- | --- | --- |
|  | **Favourable** | **Medium** | **Unfavourable** | **Ptrend** | **Favourable** | **Medium** | **Unfavourable** | **Ptrend** |
| **Weighted SDH score range** | [0–2.84] | (2.84–4.91] | (4.91–14] |  | [0–4.2] | (4.2–6.6] | (6.6–9.0] |  |
| Number of participants | 16,442 | 16,158 | 16,297 |  | 2,601 | 2,649 | 2,521 |  |
| **Cardiovascular disease mortality** |  |  |  |  |  |  |  |  |
| HR (95% CI) in Model 1 | 1 (ref) | 1.18 (0.78-1.79) | 3.31 (2.33-4.70) | 7.00E-12 | 1 (ref) | 2.17 (1.42-3.30) | 3.04 (2.00-4.65) | 4.20E-08 |
| HR (95% CI) in Model 2 | 1 (ref) | 1.08 (0.72-1.64) | 2.55 (1.78-3.65) | 7.10E-08 | 1 (ref) | 1.92 (1.26-2.91) | 2.35 (1.52-3.62) | 1.50E-04 |
| **Cancer mortality** |  |  |  |  |  |  |  |  |
| HR (95% CI) in Model 1 | 1 (ref) | 1.09 (0.87-1.37) | 1.84 (1.49-2.26) | 7.20E-09 | 1 (ref) | 1.10 (0.74-1.64) | 1.52 (1.03-2.24) | 2.10E-02 |
| HR (95% CI) in Model 2 | 1 (ref) | 1.04 (0.83-1.31) | 1.55 (1.25-1.91) | 3.10E-05 | 1 (ref) | 1.00 (0.68-1.49) | 1.29 (0.87-1.92) | 1.50E-01 |

Note: Model 1 was adjusted for age and sex; Model 2 was additionally adjusted for BMI, smoking status, drinking status, diet, physical activity, prevalence of hypertension and diabetes.

Abbreviations: SDH, social determinant of health; NHANES, National Health and Nutrition Examination Survey.

# Supplementary methods

# Measurements of covariates

A range of important covariates were collected in this analysis, including age, sex (male and female), body mass index (BMI), lifestyle behaviors (smoking status, drinking status, physical activity and diet), and history of hypertension and diabetes.

Specifically, smoking status was classified as never, previous or current. In US NHANES, participants who never smoked 100 or more cigarettes in their lifetime were defined as never smokers; participants who smoked 100 or more cigarettes in the past but currently do not smoke were defined as previous smokers; and participants who smoked 100 or more cigarettes and reported daily smoking in the past 30 days were defined as current smokers.

Drinking status was classified as never, previous or current. In US NHANES, participants who never consumed or did not consume 12 or more alcohol-based drinks in the past year were defined as never drinkers; participants who consumed 12 or more drinks in their lifetime but not in the past year were defined as previous drinkers; and participants who consumed 12 or more drinks in the past year with reported weekly intake were defined as current drinkers.

Physical activity was categorized into three groups: inactive, insufficiently active, and active. In the UK Biobank, physical activity was defined based on the sum of days spent performing walking, moderate, and vigorous activities. Participants were then divided into inactive, insufficiently active, and active groups using tertiles of physical activity. In the US NHANES, participants with no leisure-time physical activity were classified as inactive; participants engaging in leisure-time moderate activity 1-5 times per week, leisure-time vigorous activity 1-3 times per week, or those with metabolic equivalents less than 6 were classified as insufficiently active; and participants engaging in more leisure-time moderate or vigorous activity than these criteria were classified as active.

Diet was classified as healthy diet and unhealthy diet based on Healthy Eating Index (HEI) in US NHANES and a dietary recommendation according to a previous study in UK Biobank [1, 2]. In UK biobank, dietary diversity score was obtained from 24-hour dietary recalls and based on a dietary recommendation according to a previous study [1]. Five items of the recommendations were included, including grain products (whole grains and non-whole grains), Vegetables (dark green leafy, vitamin A-rich, starchy tubers, and other), Fruits (citrus, vitamin A-rich and other), Meat and protein alternatives (red meat, poultry, fish and seafood, organ meat, eggs and legumes, and nuts), and Dairy products (milk, yoghurt and cheese). We calculated the average value of repeated diet evaluations. For each large category, if a participant consumes any food in this category, the score will increase. However, in the same small group, different diverse food intakes will not be repeatedly calculated. Therefore, the dietary diversity score ranged from 0 to 18. In US NHANES, dietary quality was obtained from 24-hour dietary recalls and was assessed by healthy eating index (HEI) scores [2]. Diet was divided into unhealthy diet (below the median) and healthy diet (above the median) through median of diet score.

The prevalence of hypertension was defined based on ICD-10 codes from I10 to I50 in UK Biobank. In US NHANES, hypertension was identified based on one or more of these conditions: the use of antihypertensive medication, systolic blood pressure (SBP) ≥140 mmHg, diastolic blood pressure (DBP) ≥90 mmHg, or an affirmative response to "Ever told you had high blood pressure".

Diabetes was diagnosed through ICD-10 codes E10 to E14 in UK Biobank. In US NHANES, diabetes was defined based on one or more of these conditions: glycosylated hemoglobin (HbA1c) ≥6.5%, the current use of diabetes medication or insulin, or an affirmative response to the question "doctor told you have diabetes".

The detailed information of covariates was documented in Supplementary table S4.

**References:**

1. Zheng, G., et al., *Dietary Diversity and Inflammatory Diet Associated with All-Cause Mortality and Incidence and Mortality of Type 2 Diabetes: Two Prospective Cohort Studies.* Nutrients, 2023. **15**(9).

2. Li, Y., et al., *Healthy lifestyle and life expectancy free of cancer, cardiovascular disease, and type 2 diabetes: prospective cohort study.* Bmj, 2020. **368**: p. l6669.
